# Orthodontic treatment as an adjunct to periodontal therapy

**DOI:** 10.1590/2177-6709.24.4.080-092.bbo

**Published:** 2019

**Authors:** Pedro Marcelo Tondelli

**Affiliations:** 1 Universidade Estadual de Londrina, Departamento de Medicina Oral e Odontologia Infantil (Londrina/PR, Brazil). Universidade Estadual de Londrina, Curso de Especialização em Ortodontia (Londrina/PR, Brazil).

**Keywords:** Malocclusion, Tooth movement, Orthodontics, Periodontitis

## Abstract

This study discusses the role of orthodontic treatment as an adjunct to the control and treatment of periodontal disease conditions, and describes a clinical case of severe anterior mandibular crowding and periodontal disease followed up for nine years and three months after orthodontic treatment completion. Malocclusion impaired proper dental hygiene, which led to bone loss and development of a periodontal abscess between mandibular canines and lateral incisors. After scaling and root planing, orthodontic treatment was initiated with extraction of the four second premolars, to correct the deficiency detected in cephalometric and model analysis. Treatment objectives were met, and facial and dental esthetics was satisfactory. Adequate periodontal management, hygiene control and tooth movement ensured ideal occlusion and facilitated the control of biofilm.

## RESUMO

Os objetivos do presente artigo são discutir o papel do tratamento ortodôntico como coadjuvante no controle e tratamento dos problemas periodontais e apresentar um caso clínico com severo apinhamento anteroinferior e problemas periodontais, com acompanhamento de 9 anos e 3 meses após o tratamento ortodôntico. A má oclusão impossibilitava a correta higienização dos dentes, causando perda óssea e abscesso periodontal entre os caninos e incisivos laterais inferiores. Após a raspagem e alisamento radicular, o tratamento ortodôntico foi realizado com extrações dos quatro segundos pré-molares, para corrigir a discrepância cefalométrica e de modelos. Os objetivos do tratamento foram alcançados com satisfatória estética facial e dentária. O adequado manejo do periodonto, controle da higiene bucal e da movimentação dentária permitiram atingir uma oclusão ideal e facilitar o controle de placa bacteriana.

## INTRODUCTION

Orthodontic treatments are based on tooth movement, which is possible because of the periodontal ligament, which unites tooth roots to the alveolar bone. The forces applied to a tooth produce stresses on the periodontal ligament, with areas of traction or stretching that induce bone formation; and areas of compression that promote bone resorption and, consequently, tooth movement.^1^ In this process, a healthy periodontium is essential to avoid any compromise to tooth-supporting tissues.^2^

The primary etiological factor of the development of periodontal diseases is the pathogenic microflora on dental biofilm, in close contact with gingival margins.^3^ ^,^ ^4^ Gingivitis is a moderate form of periodontal disease, not associated with periodontal attachment loss. However, a change in the balance between the biofilm and the host may lead to periodontitis, an intensification of the disease that is associated with bone loss.^4^ Therefore, the balance between this microflora and the host’s immune response is fundamental to control periodontal disease, and the main objective of periodontal treatment is biofilm disruption and removal.^5^

Orthodontic treatments may contribute to periodontal health, because they align teeth and balance occlusion, which improves hygiene, as it facilitates access to teeth and reduces occlusal trauma.^6^ However, fixed orthodontic appliances may increase supragingival biofilm accumulation and deteriorate periodontal health.^2^ The characteristics of tooth surfaces may affect the quality and quantity of accumulated biofilm,^7^ which may lead to an increase in the amount of pathogenic anaerobic bacteria in supra- or subgingival biofilm during orthodontic treatment. Because of that, the disease should be controlled by means of adequate hygiene.^8^

The control of periodontal disease and biofilm formation allows that patients with this disease receive orthodontic treatment to correct malocclusion and tooth crowding, factors that may contribute to this condition. Moreover, orthodontic treatment promotes root parallelism, which adequately distributes occlusal forces and corrects vertical bone defects.^9^ In case of poor periodontal attachment due to bone loss, there are changes in the crown-root ratio, and the fulcrum of the movement is apically displaced, which intensifies the load in this region and increases the chances of root resorption.^10^ Smaller and thinner roots have a thinner periodontal ligament, and forces are, thus, concentrated on a smaller area.^11^ More extensive movements may, therefore, result in greater risk of root resorption. 

Six factors^12^ are seen as benefits of orthodontic treatment of patients with periodontal disease:

1. Alignment of crowded anterior teeth, improving access to all tooth surfaces during hygiene, which is a great advantage for patients that are prone to bone loss or that do not have the manual dexterity necessary to maintain good oral hygiene.
2. Tooth uprighting, which may correct certain bone defects and often rules out the need for osteotomy.
3. Esthetic improvement of coronal positioning before restoration, which may eliminate the need for gingival recontouring, a procedure that may require bone excision and root exposure.
4. Teeth with fracture, perforations, subgingival or intraosseous caries may be treated with adequate restorations or prostheses after forced eruption, which may even improve resistance and retention.
5. Elimination of open embrasures, which affect esthetics in the anterior region, and may be corrected by tipping the roots of adjacent teeth or by reducing interproximal distance or distance between roots.
6. The position of adjacent teeth may be improved before implants, fixed or removable prostheses are placed.

Thus, this study discusses the role of orthodontic treatment as an adjunct to the control and treatment of periodontal disease, and describes a clinical case of severe anterior mandibular crowding and periodontitis followed up for nine years and three months after completion of orthodontic treatment.

## CASE REPORT

A 36-year and 6-month-old man was referred by his general dentist because of periodontitis in the anterior mandibular region, with abscesses and horizontal bone loss. The patient complained that severe crowding complicated his oral hygiene and exacerbated the problem. 

According to his history, he was in good health and had no medical problems. Clinical examination revealed satisfactory oral hygiene and no caries or restorations, and the patient’s compliance with initial oral hygiene instructions was good.

## DIAGNOSIS

Facial analysis revealed a symmetrical frontal aspect, except for a nasal septal deviation to the right. His profile was slightly convex, and his lower anterior facial height was increased.

**Figure 1:**
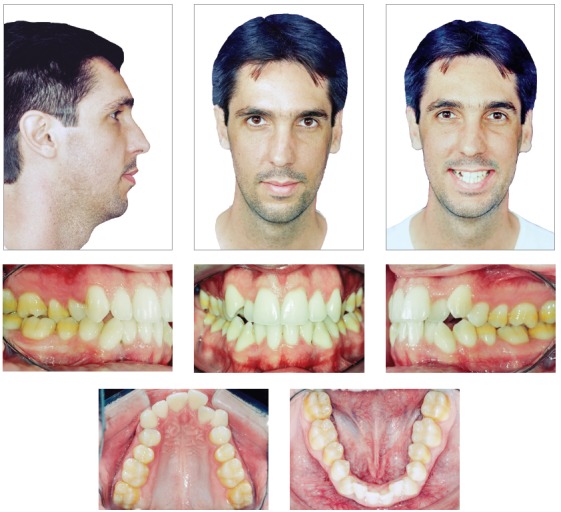
Initial facial and intraoral photographs.

Intraoral examination confirmed Class I malocclusion and an 8-mm crowding in both arches, deficiency in the region of maxillary premolars, crossbite of left maxillary premolars and transversal edge-to-edge relationship of left maxillary first molar. Upper midline was 1 mm to the right, and the right maxillary lateral incisor (tooth #12) was positioned lingually (Fig 1).

Canine guidance in lateral excursion was absent, because there were no contacts between maxillary and mandibular canines. Mandible examination did not reveal any differences between centric relation (CR) and habitual occlusion (HO). 

**Figure 2:**
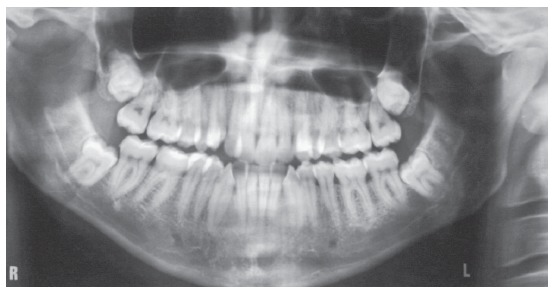
Initial panoramic radiograph.

**Figure 3:**
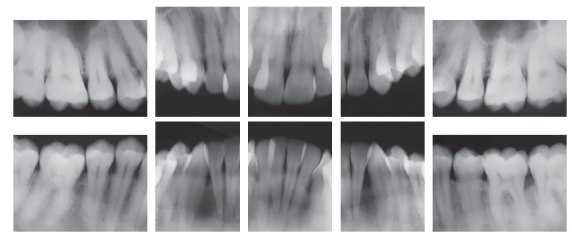
Initial periapical radiographs.

The initial panoramic radiograph showed normal bone and dental structures, with low mineral density of bone crest between mandibular canines and lateral incisors, and unerupted and impacted third molars (Fig 2). Periapical radiographs showed a periodontal disease condition between mandibular canines and lateral incisors, with bone loss and low density extending to the middle third of the roots, which is characteristic of a periodontal abscess (Fig 3).

**Figure 4:**
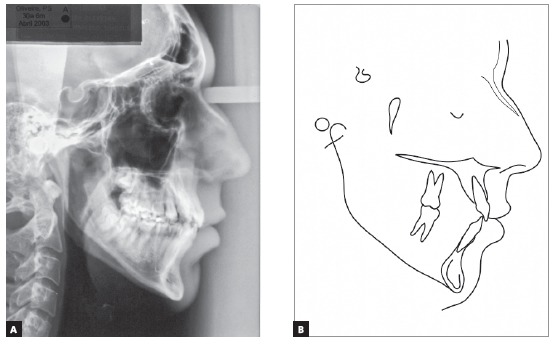
Initial cephalometric profile radiograph (A) and cephalometric tracing (B).

Cephalometric analysis (Figs 4A and 7B, Table 1) revealed a Class I skeletal pattern (ANB = 4.5^o^). 

**Table 1:** Cephalometric measurements at baseline (A), treatment completion (B) and nine years and three months after treatment completion (C).

	Medidas		Normal	A	B	C	Dif. A/B
Skeletal pattern	SNA	(Steiner)	82°	81.5°	81°	81°	0.5
	SNB	(Steiner)	80°	77°	78°	78°	1
	ANB	(Steiner)	2°	4.5°	3°	3°	1.5
	Wits	(Jacobson)	♀ 0 ± 2 mm ♂ 1 ± 2 mm	2mm	- 1.5mm	- 1.5mm	3.5
	Angle of convexity	(Downs)	0°	4.5°	3°	3°	1.5
	Y-axis	(Downs)	59°	61°	61°	61°	0
	Facial angle	(Downs)	87°	87°	87°	88°	0
	SN-GoGn	(Steiner)	32°	40°	38°	38°	2
	FMA	(Tweed)	25°	31°	31°	31°	0
Dental pattern	IMPA	(Tweed)	90°	87°	81°	81°	6
	1.NA (degrees)	(Steiner)	22°	18°	19°	19°	1
	1-NA (mm)	(Steiner)	4 mm	5mm	4mm	4mm	1
	1.NB (degrees)	(Steiner)	25°	22°	17°	17°	5
	1-NB (mm)	(Steiner)	4 mm	9mm	5mm	5mm	4
	- Interincisal angle	(Downs)	130°	134°	140°	140°	6
	- Apo	(Steiner)	1mm	4mm	1.5mm	1mm	2.5
Profile	Upper lip - S-line	(Steiner)	0 mm	2mm	- 1.5mm	- 2mm	3.5
	Lower lip - S-line	(Steiner)	0 mm	4mm	- 2mm	- 2mm	6

The SN.GoGn (40^o^), FMA (31^o^) and Y axis (61^o^) angles confirmed that his facial pattern was vertical. The position of maxillary incisors was normal, with a reduced axial inclination (1.NB = 22^o^, IMPA = 87^o^), and mandibular teeth had a satisfactory inclination (1.NB = 22^o^, IMPA = 87^o^), but were protruded (1-NB = 9 mm). The interincisal angle was close to normal (134^o^), and upper and lower lips were protruded, according to Steiner’s S line (2 mm and 4 mm).

## TREATMENT OBJECTIVES

The objectives of the treatment were the control of oral hygiene, by providing adequate instructions about brushing and flossing, and, above all, the control of periodontal disease in the mandibular anterior region. Other objectives were tooth alignment, to facilitate brushing and flossing, and to contribute to the improvement of the periodontal condition. This case required follow-up and previous and concomitant interventions by a periodontist for the treatment of periodontitis. 

The extraction of maxillary and mandibular premolars would provide the space necessary to correct crowding, reposition incisors and consequently improve facial profile. Second premolars were an adequate choice for extraction, because they had a dark band in the center of their clinical crowns, highly compromising smile esthetics. Their extraction would also favor vertical control of orthodontic mechanics by means of loss of posterior anchorage, as the patient had a dolichocephalic facial profile. 

Despite extractions, the arches would still have to be enlarged, particularly at the level of premolars, were they were constricted, which was expected to improve smile esthetics and decrease the buccal corridor on both sides.

## TREATMENT PLAN

The treatment plan consisted of banding maxillary first and second molars using bands with tubes for extraoral appliance (EOA) and placing a transpalatal arch (TPA) between maxillary first molars. Treatment would continue with the extraction of maxillary and mandibular second premolars, and the direct bonding of standard Edgewise brackets (0.022 x 0.028-in slot, 3M-Abzil) to premolars and canines. 

First premolars and canines would be moved distally using 0.016-in stainless steel segmented arches with teardrop loops, to obtain space for bonding and incisor alignment. After space was obtained, alignment and leveling would be conducted using 0.0155-in, 0.0175-in (both coaxial), 0.016-in, 0.018-in, 0.020-in, 0.017 x 0.025-in and 0.019 x 0.025-in stainless steel wires.

After alignment and levelling, the canines would be moved 3 mm distally using 0.019 x 0.025-in stainless steel wires. In the posterior area, retraction archwires with teardrop loops distal to lateral incisors would be used for the retraction of mandibular and maxillary incisors, because a 3-mm repositioning of incisors with controlled tipping would improve lip posture and facial profile.

Finishing would consist of using maxillary and mandibular 0.019 x 0.025-in ideal archwires together with intermaxillary elastics. 

Maxillary retention would be performed with a removable wraparound acrylic retainer, involving all maxillary teeth until maxillary second molars, and a maxillary anterior lingual retainer (3 x 3, using a 0.7-mm wire) bonded to the canines.

Third molar extraction would be recommended at the end of treatment.

## TREATMENT PROGRESSION

**Figure 5:**
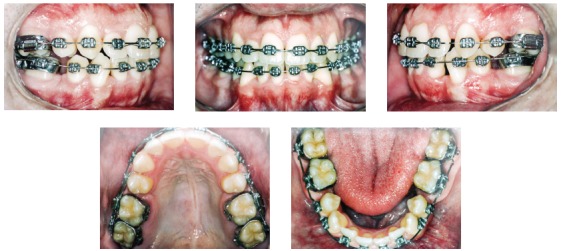
Intermediate intraoral photographs after distal movement of canines and alignment of incisors.

The greatest challenges of this treatment were periodontal control by the patient, closing extraction spaces, as the patient was an adult, and keeping vertical control. The treatment followed the initial plan described above. After retraction of mandibular incisors and closure of all extraction spaces, 0.019 x 0.025-in ideal archwires were used, with 5/16-in medium-force intermaxillary elastics in the region of premolars and molars (Fig 5).

## TREATMENT RESULTS

**Figure 6:**
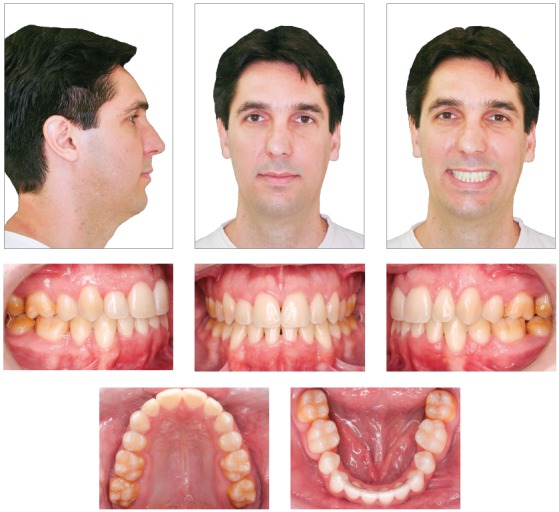
Final facial and intraoral photographs.

Facial esthetics improved because of a more adequate lip posture. The lips followed the lingual tipping of incisors, which decreased facial convexity (Fig 6).

Treatment maintained the Angle Class I molar relationship and achieved good intercuspation of premolars and canines. At the end of the treatment, midlines were coincident. Dental arch form and the position of teeth in relation to basal bones were satisfactory (Fig 6).

**Figure 7:**
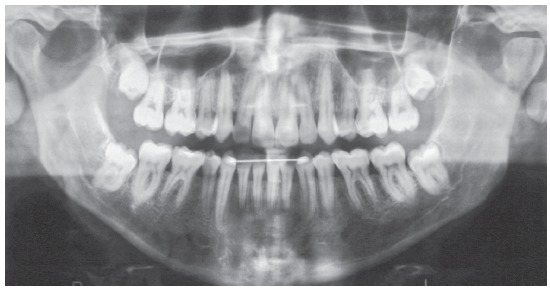
Final panoramic radiograph.

**Figure 8:**
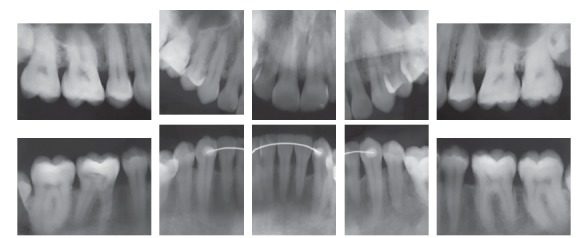
Final periapical radiographs.

**Figure 9:**
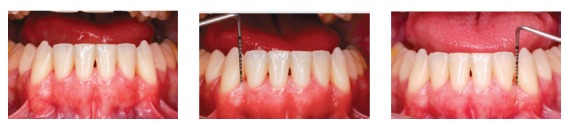
Mandibular incisor photographs showing probing and confirming periodontal health.

The analysis of panoramic radiographs revealed that the tooth roots were parallel at the end of the treatment. Third molars were unerupted, and their extraction was indicated (Fig 7). Periapical radiographs showed that the roots were preserved during treatment, and there was no additional bone loss between mandibular lateral incisors and canines (Fig 8). Periodontal probing confirmed that the periodontium was healthy (Fig 9).

**Figure 10:**
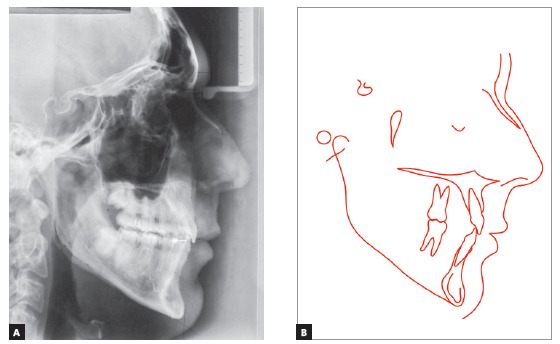
Final cephalometric profile radiograph (A) and cephalometric tracing (B).

Cephalometric analysis showed that lower anterior facial height was maintained (FMA = 31^o^ and Y axis = 61^o^), although SN.GoGn (38^o^) had a 2-degree reduction, and the ANB angle (3^o^), a 1.5-degree reduction. Mandibular incisors were repositioned lingually (IMPA = 81^o^, 1-APo = 1.5 mm and 1-NB = 5 mm), with controlled tipping of incisors, whose apices were centralized at the symphysis, confirmed by the 5-degree reduction of the 1.NB angle (17^o^). Maxillary incisors were retruded, and their position improved, confirmed by the 1-mm reduction of 1-NA (4mm) and the 1-degree increase of 1.NA (19^o^), which contributed to the increase of interincisal angle (140^o^) (Fig 10A, Table 1).

**Figure 11:**
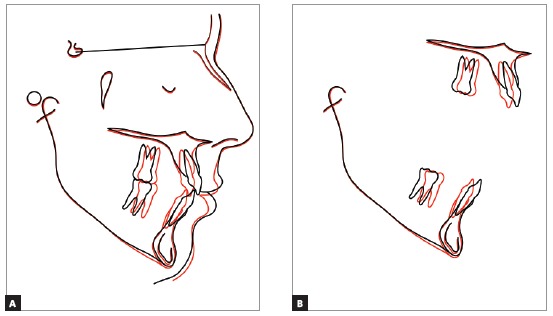
Total (A) and partial (B) superimpositions of baseline (black) and final (red) cephalometric tracings.

Total cephalometric superimposition confirmed the results expected for an adult patient, without facial growth: Maxillary and mandibular incisors were retracted, and the lips were repositioned lingually, which improved the patient’s profile. Lower anterior facial height did not change, as the mandibular plane was preserved (Fig 11A). 

Partial superimposition showed the mesial movement of maxillary and mandibular first molars, the repositioning of mandibular incisors by means of controlled tipping and preservation of the apex inside the symphysis, and the bodily retraction of maxillary incisors, controlling torque (Fig 11B). 

## FOLLOW-UP AFTER TREATMENT COMPLETION

**Figure 12:**
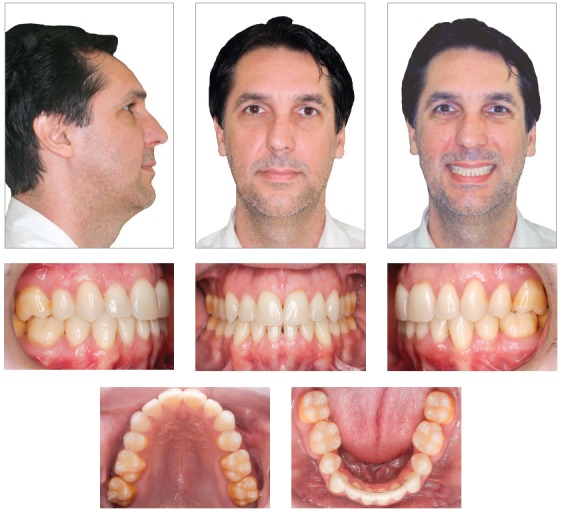
Follow-up facial and intraoral photographs nine years and three months after treatment completion.

**Figure 13:**
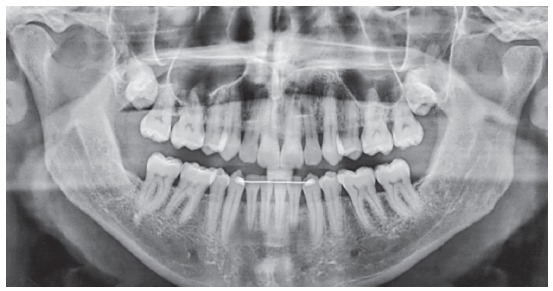
Follow-up panoramic radiograph nine years and three months after treatment completion.

**Figure 14:**
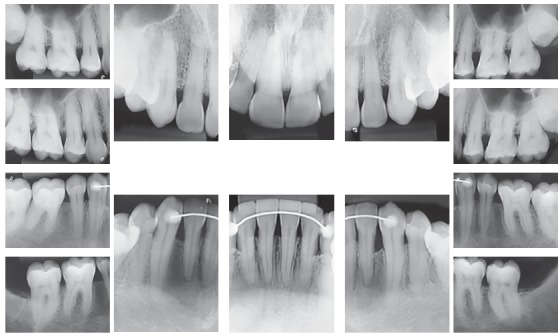
Follow-up periapical radiographs nine years and three months after treatment completion.

**Figure 15:**
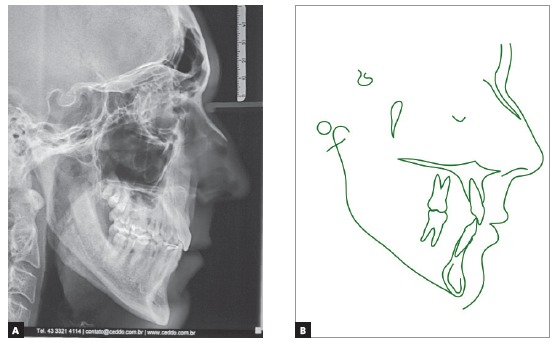
Follow-up cephalometric profile radiograph (A) and cephalometric tracing (B) nine years and three months after treatment completion.

Clinical examinations nine years and three months after treatment completion confirmed stability of tooth and arch positions, preservation of smile esthetics and ideal tooth function (Fig 12). Panoramic and periapical radiographs revealed stability of bone structures, particularly in the area of the anterior mandibular teeth that had been affected by periodontal disease (Figs 13 and 14). Cephalometric analysis confirmed the stability of the measurements made at the end of orthodontic treatment (Fig 15).

## DISCUSSION

The orthodontic treatment of adults with periodontal disease should be conducted in association with other specialties to achieve good results. A healthy periodontium basically depends on biofilm disruption and removal before, during and after orthodontic treatment^2^ ^-^ ^5^. Certain types of crowding and rotation may complicate the oral hygiene, which increases the risk of periodontal disease, leading to alveolar bone loss and compromising tooth longevity^6^. 

Orthodontic devices may complicate the control of biofilm on tooth surfaces^2^ ^-^ ^7^ and promote the accumulation of pathogenic anaerobic bacteria^8^. Therefore, patients should be made aware of the importance of oral hygiene and should be adequately trained to use toothbrushes and dental floss. Moreover, oral hygiene should be followed up, during orthodontic treatment, by the orthodontist, the general dentist or the periodontist, so that biofilm is kept under control.

In this clinical case, the extraction of the maxillary and mandibular second premolars was indicated to correct incisor crowding. Scaling and root planing of mandibular anterior teeth was conducted to remove a periodontal abscess before orthodontic treatment. Fourteen days later, brackets were bonded and distal movement of premolars was initiated using segmented 0.016-in stainless steel archwires. Treatment progression led to an improvement in oral hygiene. As the first premolars and canines moved distally into the extraction spaces, incisor alignment facilitated brushing and, mainly, flossing in the region between mandibular canines and lateral incisors. Therefore, the improvement of oral hygiene due to anterior tooth alignment, associated with follow-up and motivation to control biofilm accumulation, resulted in the control of periodontal disease, one of the major benefits of orthodontic treatment for patients with periodontal disease^12^.

Orthodontic mechanics is basically extrusive, as tooth movements begin with stress applied to the periodontal ligament, deflection of the bone and tooth extrusion^1^ ^,^ ^13^. When combined with a vertical growth pattern, this makes orthodontic correction a challenge to the achievement of ideal treatment objectives, balanced occlusion and good dental and facial esthetics^14^. In the case presented here, extractions favored the correction of tooth crowding and of cephalometric deficiencies. They also contributed to a good dental and facial esthetic outcome, as tooth positions were improved by preserving the mandibular plane and the lower anterior facial height. 

Final radiographs showed that the tooth roots were preserved, which confirmed that the use of light continuous or dissipating forces is appropriate to avoid or minimize root resorptions^1^. Periapical radiographs should be obtained before, during and after the treatment, to analyze and control bone loss and root resorption, which may be intensified by a low level of bone attachment^10^ ^,^ ^11^.

When the loops in the 0.019 x 0.025-in retraction archwire are activated without torque application or Gable bends, the center of rotation is displaced to the apex of incisors during their retraction. Controlled tipping^14^, thus obtained, is more appropriate for cases of reduced periodontium in which the center of rotation is apically displaced.^10^ Because of this control of retraction and the tipping of mandibular incisors, satisfactory overjet and overbite were obtained, and ideal dental function was achieved. At treatment completion, the patient had immediate disocclusion by canine and incisor guidance, improved lip seal and better facial esthetics. 

In this case, orthodontic treatment changed tooth alignment and facilitated proper access to teeth for adequate oral hygiene using dental floss and toothbrush. This improved and preserved periodontal health, the main reason why orthodontic treatment was recommended for this patient. His condition was maintained nine years and three months after the end of the treatment.

## CONCLUSION

Orthodontic treatment is an important adjunct in the treatment and control of periodontal disease, as it promotes the correct management of the periodontium and facilitates oral hygiene and biofilm control. Treatment objectives were achieved in this case, and the results were satisfactory for dental and facial esthetics. Although the vertical growth pattern associated with an unfavorable periodontal condition determined an uncertain treatment prognosis, ideal occlusion and favorable esthetic results were achieved. 
